# Research Hotspots and Emerging Trends in Osteoporosis Epigenetics

**DOI:** 10.1155/genr/7389868

**Published:** 2026-05-20

**Authors:** Maobin Zhou, Honglei Xiao, Ang Li, Hongze Chang, Xiaolong Yang, Feng Cai, Xiaodong Liu

**Affiliations:** ^1^ Department of Orthopedics, Yangpu Hospital, School of Medicine, Tongji University, Shanghai, 200090, China, tongji.edu.cn

**Keywords:** artificial intelligence, epigenetics, estrogen, expression, mesenchymal stem cells, osteoporosis

## Abstract

**Background:**

Osteoporosis (OP) and its associated complications have emerged as critical public health challenges worldwide, primarily due to the high rates of disability and mortality they cause. In terms of its complex pathogenesis, epigenetic mechanisms play significant roles. Against this backdrop, this study aims to clarify the current research trends and hotspots in OP.

**Methods:**

Literature was retrieved from the “Web of Science,” “PubMed,” and “Scopus.” Subsequently, publication counts, country distribution, regional collaborations, and keyword analyses were conducted by using the “Bibliometrix” and “ggplot2” packages in *R* Studio. For visualization analysis, institutional cooperation and keyword relationships were examined using “CiteSpace” and “VOSviewer.”

**Results:**

A total of 464 publications were included in this analysis. The five most frequently cited keywords were “postmenopausal osteoporosis (PMOP),” “expression,” “mesenchymal stem‐cells (MSCs),” “osteogenic differentiation,” and “differentiation.” Keyword co‐occurrence analysis further identified “expression,” “PMOP,” and “MSCs” as predominant research themes. Moving forward, the findings suggest that future research will focus on elucidating the role of miRNAs in the epigenetic mechanisms of OP and identifying potential therapeutic targets. Regarding global collaboration, China led in international partnerships, particularly with the United States.

**Conclusion:**

Epigenetic mechanisms constitute a core regulatory factor in OP, serving as the critical link between genetic predisposition and environmental influences. Emerging research emphasizes the combined analysis of epigenetics and metabolomics, artificial intelligence (AI)–based prediction of epigenetic regulatory networks, and gene editing therapies targeting epigenetic factors. Furthermore, therapeutic strategies—including gene therapy, stem cell–based interventions, and small‐molecule targeted compounds—represent pivotal future directions for OP therapeutics.

## 1. Introduction

The increasing aging of the global population has significantly increased the incidence of musculoskeletal degenerative diseases. Among them, osteoporosis (OP) has become a major public health challenge due to its high disability and mortality rates. Epidemiological data show that among individuals aged over 50 years worldwide, the prevalence of OP in women is approximately 33.3% and 25.0% in men. Notably, the mortality rate is higher in male patients after fractures [1, 2]. Characterized by reduced bone density and compromised microarchitecture, OP increases bone fragility and fracture susceptibility. This condition predominantly affects the lumbar spine and hip; consequently, even minor trauma can cause fractures, potentially resulting in permanent disability or death in severe cases [3].

The core pathological mechanisms of OP include bone resorption and formation imbalance, stem‐cell exhaustion, and metabolic dysfunction [4]. Crucially, epigenetic regulation serves as a pivotal regulatory factor of osteoporotic pathogenesis, thereby offering significant guidance for OP research and treatment [5]. Recent studies reveal the contributions to OP‐related signaling pathways of epigenetic mechanisms, notably DNA methylation and histone modifications. Importantly, these mechanisms also highlight the inherent reversibility of epigenetic modifications, collectively providing a variety of insights into the pathogenesis and therapeutic interventions of OP [6]. Given these advances, it is essential to summarize current research hotspots and emerging trends while equally prioritizing the clarification of future epigenetic‐based therapies alongside the continued analysis of gene regulation.

Bibliometrics, a scientific evaluation methodology based on quantitative analysis, statistically examines academic literature to reveal research trends, core themes, and academic influence in specific fields [7]. When applied to the epigenetics of OP, this approach facilitates a systematic assessment of diagnostic potential and mechanistic insights. Moreover, by integrating multiomics data with machine learning algorithms, bibliometric analysis comprehensively maps the current research landscape and identifies key focal points in the field.

## 2. Materials and Methods

### 2.1. Data Search Strategy

This study employed a systematic literature search strategy to comprehensively synthesize research on epigenetics and OP. The data sources included the “Web of Science,” “PubMed,” and “Scopus” databases, covering publications from December 2005 to April 2025—a 20‐year span selected to trace the evolution of global research. The literature search across all three databases was performed with a cutoff date of April 30, 2025, to ensure the synchronization of journal data for the included articles. Search queries combined subject headings with free‐text terms, utilizing core terminology such as “epigenetics,” “osteoporosis,” and so on (full search strategies are detailed in Supporting File 1). Boolean logical operators (AND, OR) were integrated to construct compound search strings. Additionally, the Medical Subject Headings (MeSH) thesaurus was applied to standardize and expand the search terms, thereby ensuring comprehensiveness and precision in the retrieval process.

### 2.2. Data Collection and Screening

**FIGURE 1:**
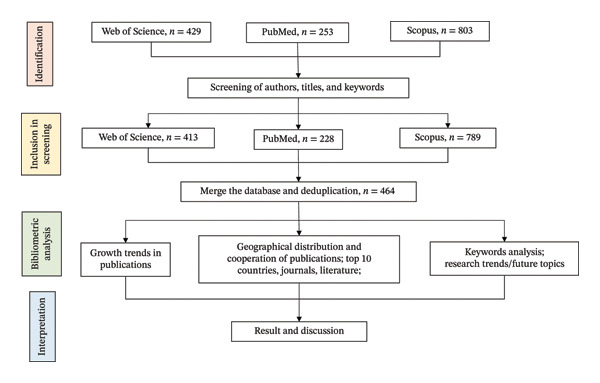
The flowchart of bibliometric analysis in osteoporosis epigenetics.

Duplicate removal was performed using EndNote X9 software. After automated deduplication by the software, two researchers specializing in OP conducted a comprehensive manual review of all pooled records to verify the accuracy of duplicate removal and to identify any potential merging errors. Following application of the inclusion and exclusion criteria to screen the literature, 464 articles were ultimately selected for subsequent analysis (Figure 1). The inclusion criteria were as follows: data obtained from the “Web of Science,” “PubMed,” and “Scopus” databases; explicitly involving epigenetic mechanisms and related to OP; limited to original studies or reviews; and a data cutoff date of April 2025. The strategy of including both original research articles and reviews aims to focus the analysis on high‐quality, peer‐reviewed literature that represents key consensus and progress in the field of OP. Conversely, the exclusion criteria comprised the following: data that did not involve epigenetic mechanisms or were not related to OP; type of literature such as conference abstracts and case reports; literature not in English; and duplication of studies or incomplete data. To ensure dataset relevance and scientific rigor, further irrelevant literature was eliminated. Throughout this screening process, titles, abstracts, and keywords were manually reviewed.

### 2.3. Data Analysis

This study adopted an integrated methodology, combining bibliometric and visual analysis techniques. Initially, a comprehensive dataset was extracted, such as keywords, publication volume, publication year, country/region of origin, journal sources, highly cited publications, and journal impact metrics. This data extraction delineated research trends and identified core academic strengths in the epigenetics of OP. Subsequently, the retrieved bibliographic records were imported into VOSviewer (Version 1.6.11) for further processing. Finally, data analysis and visualization were performed using the “Bibliometrix” and “ggplot2” packages in *R* software.

## 3. Results

### 3.1. Annual Publications in Epigenetics Research on OP

**FIGURE 2:**
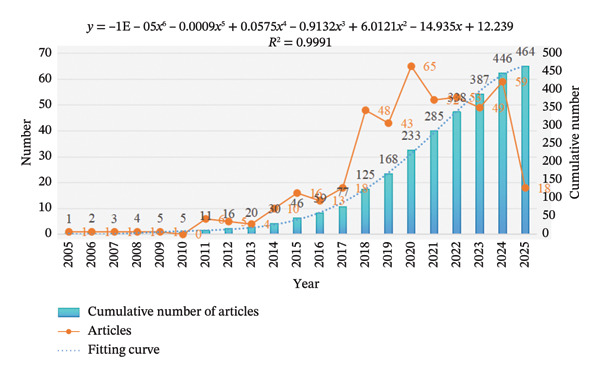
Publish trends and article numbers in “Web of Science core collection,” “PubMed,” and “Scopus” from 2005 to 2025.

Analysis of annual publication trends derived from the “Web of Science,” “PubMed,” and “Scopus” revealed a significant global increase in research output in this field between 2014 and 2024. Notably, the publication volume peaked in 2020. Over the past 5 years, the number of publications has continued to grow steadily, indicating that epigenetics research on OP has always been a hot topic (Figure 2).

### 3.2. Analysis of Research Hotspots and Frontiers

**FIGURE 3:**
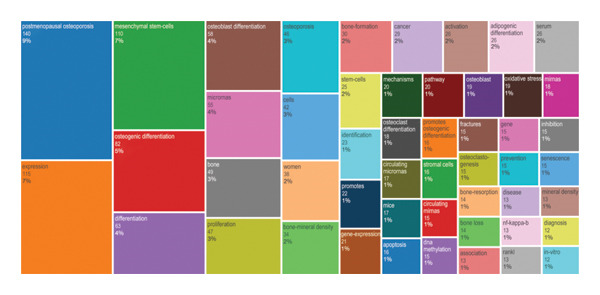
Description of keywords. Word cloud map of osteoporosis epigenetics.

Keyword co‐occurrence analysis facilitates the identification of research directions and hotspots in OP. Following keyword screening and co‐occurrence analysis, our analysis identified the five most frequently occurring keywords: “postmenopausal osteoporosis (PMOP)” (140 frequency, 9% of total mentions), “expression” (115 frequency, 7% of total mentions), “mesenchymal stem‐cells (MSCs)” (110 frequency, 7% of total mentions), “osteogenic differentiation” (82 frequency, 5% of total mentions), and “differentiation” (63 frequency, 4% of total mentions) (Figure 3).

OP research was mainly characterized by three focal areas in the early stage: therapeutic applications of MSCs (48.3%), investigation of PMOP (46.8%), and exploration of epigenetic mechanisms (41.3%) (Figures 4(a) and 4(b)). In contrast, recent research indicates a significant shift toward gene expression studies (58.7%), reflecting a deeper exploration into how epigenetic modifications—such as DNA methylation, histone modifications, and noncoding RNAs—orchestrate the transcriptional and posttranscriptional programs underlying OP pathogenesis. Concurrently, sustained research efforts continue to focus on PMOP (53.2%) and MSC‐based therapies (51.7%).

FIGURE 4(a) Colinear network of keywords; (b) trends of the top three keywords.

**Figure:**
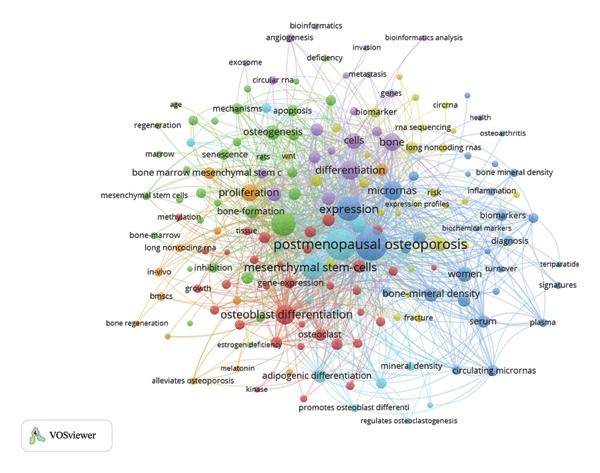
(a)

**Figure:**
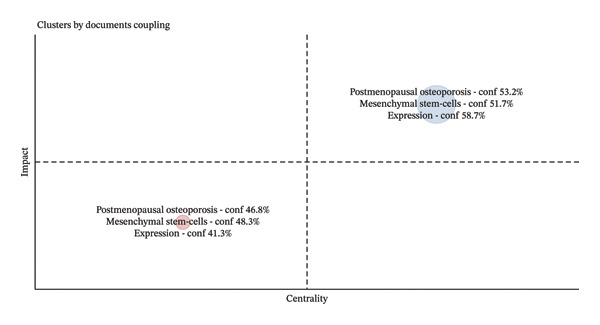
(b)

**FIGURE 5:**
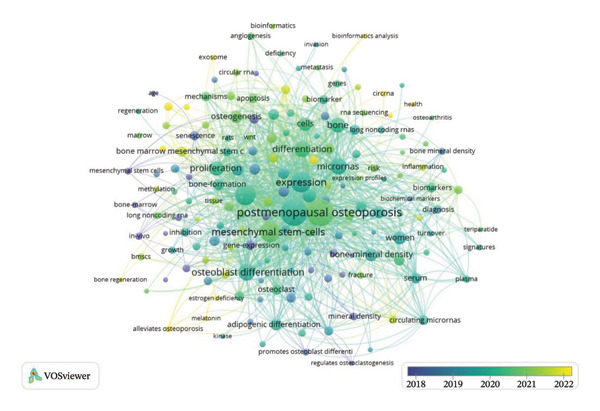
The trend of keywords over 2018–2022.

Furthermore, keyword co‐occurrence analysis spanning 2018–2022 revealed distinct evolutionary trends in research hotspots. During the initial phase (2018–2020), research largely concentrated on core areas, including “PMOP,” “MSCs,” and “expression.” However, recent dynamics (2021–2022) show a marked shift, with “bone degeneration,” “circRNA,” and “bioinformatics analyses” emerging as prominent research areas (Figure 5).

### 3.3. Country/Regional Distribution and Research Focus

Research related to OP has been conducted across 30 countries. Analysis of publication origins reveals China, the United States, and Italy as the leading contributors. It specifically shows the top 10 countries in terms of the total number of articles, with China (334 papers), the United States (33 papers), and Italy (20 papers) ranking in the top three (Table 1). Regarding regional cooperation, China exhibits the strongest collaborative ties, particularly with the United States and Australia (Figure 6(a)). In contrast, collaboration among other countries is significantly less extensive. Visualized through a co‐occurrence network, the intensity of collaboration between country pairs is indicated by the thickness of connecting lines, while distinct colors represent countries positioned along the diagram periphery.

**TABLE 1:** Top 10 countries/regions ranked by publications.

Country	Articles	SCP	MCP	MCP_ratio
China	334	311	23	0.069
USA	33	21	12	0.364
Italy	20	16	4	0.2
Germany	8	6	2	0.25
France	7	6	1	0.143
India	7	7	0	0
Japan	7	4	3	0.429
Austria	6	5	1	0.167
Iran	4	3	1	0.25
Norway	4	1	3	0.75

FIGURE 6(a) Visualization of the connection between different countries/regions; (b) visualization of the connection between different institutions, the line represents the connection, and the endpoint size represents the strength of the connection.

**Figure:**
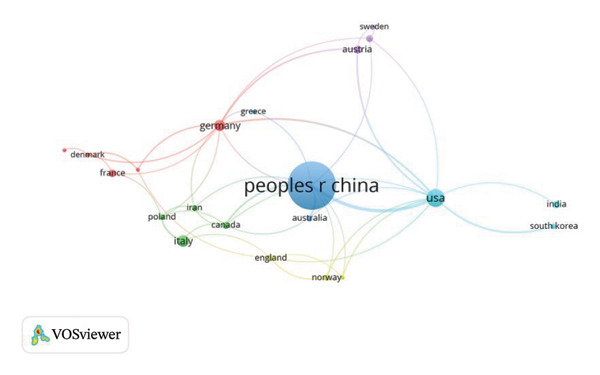
(a)

**Figure:**
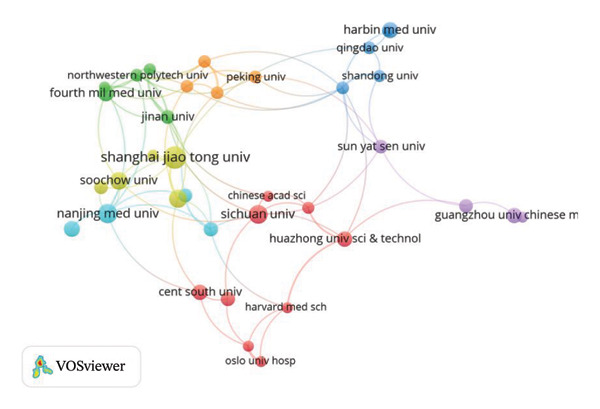
(b)

At the institutional level, publications originated from 620 distinct institutions. The VOSviewer‐generated co‐occurrence map identifies *Shanghai Jiao Tong University*, *Sichuan University, and Nanjing Medical University* as the top three contributing institutions. In addition, *Shanghai Jiao Tong University* and *the Fourth Military Medical University* exhibited closer cooperation with other institutions. Besides, interinstitutional collaboration appears to be more readily established than international collaboration (Figure 6(b)).

### 3.4. High‐Impact Journals and Articles

**FIGURE 7:**
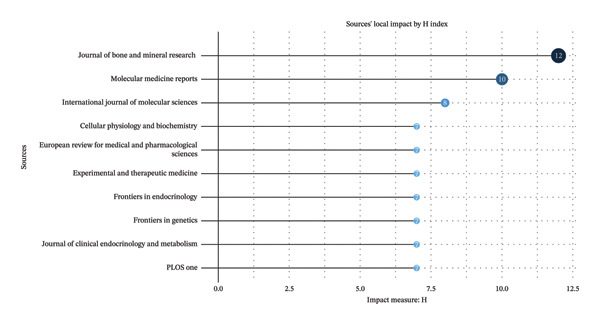
Top 10 institutions by article count and rank.

High‐quality journals and articles play a crucial role in amplifying research impact. Within this domain, *the Journal of Bone and Mineral Research* (h‐index: 12) and *Molecular Medicine Reports* (h‐index: 10) stand out as influential publications (Figure 7). Analysis of the top ten most‐cited articles revealed that 40% were published after 2016. The most frequently cited article was entitled “*MicroRNA-188 regulates age-related switch between osteoblast and adipocyte differentiation*,” published in *the Journal of Clinical Investigation* in 2015. This article exemplified how noncoding RNAs, as a key epigenetic regulator, fine‐tune gene expression networks to influence the fate of MSCs in OP [8]. Notably, 90% of the top 10 most cited articles focused on the role of microRNAs (miRNAs), highlighting this class of molecules as a significant, though not exclusive, component of the epigenetic landscape that controls posttranscriptional gene expression in OP pathogenesis (Table 2).

**TABLE 2:** Top 10 cited articles in the area of osteoporosis epigenetics.

Title	Publish year	DOI	Total citations
MicroRNA‐188 regulates age‐related switch between osteoblast and adipocyte differentiation	2015	10.1172/JCI77716	423
LncRNA MEG3 inhibited osteogenic differentiation of bone marrow mesenchymal stem cells from postmenopausal osteoporosis by targeting miR‐133a‐3p	2017	10.1016/j.biopha.2017.02.090	184
MiR‐497∼195 cluster regulates angiogenesis during coupling with osteogenesis by maintaining endothelial Notch and HIF‐1*α* activity	2017	10.1038/ncomms16003	182
The transcriptional profile of mesenchymal stem cell populations in primary osteoporosis is distinct and shows overexpression of osteogenic inhibitors	2012	10.1371/journal.pone.0045142	177
MicroRNA‐31a‐5p from aging BMSCs links bone formation and resorption in the aged bone marrow microenvironment	2018	10.1111/acel.12794	176
MiR‐503 regulates osteoclastogenesis via targeting RANK	2014	10.1002/jbmr.2032	174
Alpha‐ketoglutarate ameliorates age‐related osteoporosis via regulating histone methylations	2020	10.1038/s41467‐020‐19360‐1	162
Circulating microRNA signatures in patients with idiopathic and postmenopausal osteoporosis and fragility fractures	2016	10.1210/jc.2016‐2365	159
MiR‐133a in human circulating monocytes: a potential biomarker associated with postmenopausal osteoporosis	2012	10.1371/journal.pone.0034641	153
MicroRNA‐130a controls bone marrow mesenchymal stem cell differentiation towards the osteoblastic and adipogenic fate	2019	10.1111/cpr.12688	146

## 4. Discussion

Skeletal homeostasis critically relies on the balance between osteoblast‐mediated bone formation and osteoclast‐mediated bone resorption—a process synergistically regulated by the bone and its surrounding microenvironment [9]. Crucially, disruption of this equilibrium can activate aberrant bone repair pathways, precipitated by factors such as aging, estrogen deficiency, chronic inflammation, or metabolic disorders. Consequently, bone remodeling becomes imbalanced, characterized by increased resorption, impaired formation, and elevated marrow adiposity, ultimately leading to OP [10]. This degenerative process not only diminishes bone density but also adversely impacts the bone marrow microenvironment. Besides, it impairs the osteogenic differentiation potential of bone marrow mesenchymal stem cells (BMSCs) and establishes a vicious cycle that significantly exacerbates the fracture risk [11, 12]. Emerging evidence indicates that epigenetic mechanisms play significant roles in the dynamic regulation of bone‐related gene expression, including DNA methylation, histone modifications, and noncoding RNAs. These mechanisms profoundly affect osteogenic and lipogenic differentiation fate decisions, osteoblast activity, and osteoclast differentiation in BMSCs [13, 14]. Thus, epigenetic research on OP pathogenesis attracts increasing scientific attention.

We systematically reviewed 464 articles retrieved from the “Web of Science,” “PubMed,” and “Scopus” databases, including all epigenetic and OP‐related research published between 2005 and 2025. Analysis revealed a consistent annual increase in publication volume, with more than 50 articles published annually since 2020. This upward trend in publication highlights the ongoing momentum of research related to epigenetics in OP, indicating that academic interest in this field is continuously expanding. China, the United States, and Italy emerged as the primary contributors. In addition, while *Shanghai Jiao Tong University* has established close cooperative relationships with other institutions, the findings collectively emphasize the importance of interdisciplinary cooperation in solving complex OP‐related problems. Analysis of the top 10 journals and articles found that most publications related to OP are published in orthopedic journals, whereas interdisciplinary journals feature fewer papers. These studies primarily investigated epigenetic mechanisms, revealing the regulatory functions of miRNAs in gene expression associated with OP pathogenesis [15, 16]. Taken together, these findings emphasize the pivotal contribution of epigenetic factors to the progression of OP.

### 4.1. Major Research Directions

#### 4.1.1. Epigenetic Programming of MSCs: Directing Differentiation and Identifying Novel Therapeutic Targets

Conventional OP therapies mitigate bone loss by inhibiting the activity of osteoclasts, primarily bisphosphonates and denosumab. However, long‐term use is associated with adverse effects such as atypical femur fractures [17]. Importantly, these existing therapies fail to address the core pathological mechanism: dysregulation of MSCs in the bone marrow microenvironment [8]. Research demonstrates that the aging phenotype of bone marrow MSCs in patients with OP is closely linked to epigenetic dysregulation. This epigenetic dysregulation is particularly evident in RNA modifications, such as N6‐methyladenosine (m6A), which have emerged as crucial epigenetic regulators governing MSC proliferation, differentiation, and the maintenance of bone homeostasis [18]. Furthermore, aberrant DNA methylation silences osteogenic genes, while diminished histone acetylation promotes adipogenic program activation [19, 20]. For instance, the Bmi‐1 gene modulates H3K27me3 modification levels to concurrently suppress adipogenic differentiation and enhance the osteogenic potential of MSCs, thereby decelerating bone aging [9]. Besides, DNA methylation reprogramming targeting the Notch signaling pathway ameliorates the osteoporotic phenotype in patients with systemic lupus erythematosus by regulating Hes1 expression [21, 22]. In brief, these findings elucidate the vital regulatory role of epigenetic programming in determining MSC fate.

Current research focuses on developing precise intervention strategies targeting epigenetic regulators. Notably, epigenome editing utilizing the CRISPR‐dCas9 system has successfully activated OP‐related gene clusters in MSCs; simultaneously, in vivo studies demonstrated a more than 40% increase in bone formation rate [21]. Beyond gene editing, engineered extracellular vesicles (EVs) offer distinct advantages as natural carriers of epigenetic information. For example, MSC‐derived EVs engineered to overexpress RANK antagonize osteoclast differentiation through the delivery of specific miRNAs and significantly improve bone microstructure in animal models [23, 24]. Future advances may enable spatiotemporal‐specific epigenetic reprogramming of MSCs. Finally, it is possible to break through the single regulatory limitation of existing therapies on bone metabolism balance.

#### 4.1.2. Estrogen Deficiency Induces Epigenetic Remodeling and Bone Metabolic Imbalance

PMOP, an estrogen‐dependent metabolic bone disorder, is characterized by disrupted bone homeostasis and metabolic dysregulation. This pathology primarily arises from estrogen deficiency, which triggers bone homeostasis imbalance and epigenetic remodeling [25, 26]. According to epidemiological data, global PMOP prevalence reaches 32.1% in women over 50 years, rising sharply to 51.6% in those over 65 years. This disparity directly correlates with the abrupt decline in estrogen levels following menopausal ovarian dysfunction [27, 28].

Significantly, estrogen modulates osteoblast and osteoclast activity not only through the classical nuclear receptor pathway but also through epigenetic modifications. To illustrate, estrogen deficiency induces DNA hypomethylation in osteoclast differentiation gene promoters, while suppressing osteogenic genes through histone deacetylases (HDACs). Consequently, bone remodeling homeostasis is disrupted, leading to excessive bone resorption alongside impaired bone formation [29, 30]. Although clinical evidence confirms estrogen replacement therapy (ERT) effectively suppresses bone conversion rates, its utility remains limited by associated cardiovascular risks and an increased incidence of breast cancer [31].

Thus, targeted interventions of epigenetic mechanisms have become a research hotspot. Experimental studies have demonstrated that DNA methyltransferase inhibitors can reverse estrogen deficiency–induced adipocyte differentiation in BMSCs and restore their osteogenic capacity [32]. Additionally, selective estrogen receptor modulators exhibit tissue‐specific epigenetic regulation: They activate ERα in bone yet antagonize it in breast tissue. This selectivity, mediated by the altered recruitment of chromatin remodeling complexes, offers novel therapeutic perspectives for PMOP [33]. Recent clinical and mechanistic studies further highlight the role of specific noncoding RNAs, such as the upregulated MIR155HG/miR‐155‐5p axis in PMOP patients, in suppressing BMSC osteogenesis by targeting key regulators like DKK1 [34]. Simultaneously, research on the integration of the gut microbiota–bone axis reveals that probiotic metabolites enhance bone formation through HDAC inhibition, providing a theoretical basis for cross‐system PMOP intervention [30]. Particularly promising is the combination of stem cell transplantation with teriparatide, which accelerates fracture healing and represents a significant stride toward clinical translation [35].

#### 4.1.3. Three‐Dimensional Epigenetic Networks Integrate Environmental Interactions to Regulate Gene Expression

OP is a multifactorial disease of the skeletal system, which arises from a complex interplay between genetic variation, epigenetic regulation, and environmental factors. At the cellular and genetic levels, epigenetic mechanisms dynamically modulate the differentiation balance between osteoblasts and osteoclasts, thus influencing bone metabolic homeostasis [36]. These mechanisms extend beyond canonical DNA and histone modifications to encompass dynamic RNA epigenetic regulation. To illustrate, osteogenic differentiation of BMSCs is regulated by DNA methyltransferase 3A (DNMT3A)–mediated methylation. The abnormal expression of DNMT3A is able to inhibit the transcriptional activity of osteogenesis‐related genes and lead to diminished bone formation [9, 37]. Notably, recent evidence demonstrates that Nat10‐mediated N4‐acetylcytidine (ac4C) RNA modification enhances the translational efficiency of the key osteoclastogenic transcription factor NFATc1, thereby aggravating bone loss [38]. Also, genome‐wide association studies (GWAS) have identified over 1000 OP‐associated susceptibility loci at the genetic level. By changing the three‐dimensional chromatin conformation of distal regulatory elements or by influencing transcription factor binding, these loci can act as regulatory elements that physically interact with the promoters of critical genes like the vitamin D receptor (VDR) and low‐density lipoprotein receptor–related protein 5 (LRP5) to modulate their transcription [39]. This intricate three‐dimensional epigenetic network indicates that gene expression regulation extends beyond linear sequence information and involves the dynamic integration of chromatin spatial interactions with environmental signals [39].

Recent research has provided preliminary insights into the epigenetic regulation and transcriptional mechanisms underlying OP development. Specifically, the senescence‐associated secretory phenotype (SASP) promotes chronic inflammation in the bone marrow microenvironment by secreting proinflammatory factors. These factors suppress osteoclast differentiation through HDAC‐mediated modifications and activate osteoclastogenesis‐related pathways, such as RANKL/OPG [40, 41]. Additionally, GWAS combined with proteomics analysis found that mutations in the LRP5 gene regulate bone density through the Wnt/*β*‐catenin signaling pathway, whereas polymorphisms in the VDR gene affect calcium–phosphate homeostasis and bone matrix mineralization. Both are finely regulated by epigenetic modifications [42]. Advances in three‐dimensional genomics have further demonstrated that OP‐associated single‐nucleotide polymorphisms (SNPs) can change chromatin spatial accessibility at transcription factor binding sites, hence regulating the expression of key bone metabolism genes [39].

### 4.2. Research Hotspots

#### 4.2.1. Integrated Epigenetic–Metabolomic Analysis

With continued advances in the mechanistic study of epigenetic bone metabolism regulation, the latest evidence suggests that its research applications in OP are moving toward multidimensional innovation. Contemporary bioinformatics analyses now emphasize the central role of circRNAs in bone homeostasis regulation. Endowed with stability and tissue‐specific expression due to their closed circular structure, circRNAs serve as pivotal epigenetic elements linking genetic and environmental factors [43]. Importantly, their functions exhibit strict spatiotemporal specificity. For instance, circFam190a potently enhances osteoclast differentiation by activating the AKT1 signaling pathway, while its excessive bone‐resorptive activity is suppressible by the specific inhibitor MK‐2206—a bidirectional regulatory capacity offering a precise therapeutic target [44]. Moreover, circRNAs can trigger epigenetic cascades by modulating transcription factor nuclear translocation. To illustrate, circSTX12 regulates the proliferation of BMSCs by isolating LMO7 protein [45]. Accordingly, as a critical hinge of the epigenetic regulatory network, circRNAs represent a focal point for future breakthroughs in precision medicine for OP.

#### 4.2.2. Artificial Intelligence (AI)–Based Prediction of Epigenetic Regulatory Networks

In recent years, emerging evidence indicates that AI‐based approaches for predicting epigenetic regulatory networks have evolved into a promising research direction [46, 47]. For example, the EpiGePT model utilizes a pretrained Transformer architecture to integrate transcription factor expression with three‐dimensional genomic interaction information. This approach enables the high‐accuracy prediction of epigenomic signals and long‐range chromatin interactions across diverse cell types [48]. Besides, the HyperG‐VAE framework addresses single‐cell epigenetic heterogeneity and infers gene regulatory networks [49]. In OP research, such integrative models facilitate the characterization of regulatory targets associated with bone metabolism–related noncoding variants [50]. Importantly, AI interpretability techniques based on back‐propagation gradient analysis can identify critical regulatory network nodes; this capability provides a theoretical foundation for the screening of new drug targets [51]. In summary, AI‐driven modeling of epigenetic regulatory networks will promote the innovation of OP treatment from three dimensions: environmental response mechanisms, dynamic risk stratification, and targeted intervention strategies.

#### 4.2.3. Gene Editing Therapies Targeting Epigenetic Factors

Driven by recent advances in gene editing and tissue engineering, the deep integration of technologies such as CRISPR/Cas9 is progressively enabling spatiotemporal‐specific regulation of epigenetic reprogramming in OP treatment [52]. Therapies targeting Bmi‐1 through gene editing have demonstrated significant potential [9, 53]. In parallel, high‐throughput epigenomic studies have uncovered an additional 319 OP‐associated non‐coding susceptibility variants, among which the transcription factor YY2 modulates its target gene PAPSS2 via enhancer‐mediated chromatin looping [39]. Collectively, these advances confirm that epigenetic factor–targeted gene editing technologies represent a promising novel strategy for precise OP intervention.

## 5. Limitation

This study has several limitations. First, the limited number of publications focused on OP may lead to an underestimation of the epigenetic mechanisms involved. However, we mitigated this potential bias through the systematic and comprehensive screening of bone metabolism–related epigenetic studies. Second, our exclusive reliance on English literature may preclude the identification of regionally specific discoveries regarding epigenetic interventions for OP in non–English‐speaking regions, such as East Asia and continental Europe. Finally, certain mechanisms remain incompletely integrated into the current analysis. For example, it is still unclear how specific Traditional Chinese Medicine (TCM) compounds remodel underlying chromatin to regulate bone formation.

## 6. Conclusion

Epigenetic mechanisms play a central regulatory role in the pathogenesis of OP, crucially serving as the interface between genetic predisposition and environmental factors. This bibliometric analysis identified several key contemporary researches focuses: epigenetic programming of MSCs—directing differentiation and identifying novel therapeutic targets; estrogen deficiency induces epigenetic remodeling and bone metabolic imbalance; and three‐dimensional epigenetic networks integrate environmental interactions to regulate gene expression. Based on these emerging trends, future research is anticipated to expand into multidimensional approaches, encompassing integrated epigenetic–metabolomic analysis, AI‐based prediction of epigenetic regulatory networks, and gene‐editing therapies targeting epigenetic factors. Consequently, emerging therapeutic strategies are expected to become pivotal directions for reversing OP pathology, such as gene editing, stem cell therapy, and small‐molecule targeted drugs.

## Author Contributions

Conceptualization, data curation, formal analysis, investigation, methodology, validation, and writing–original draft: Maobin Zhou. Methodology, validation, and writing–review and editing: Honglei Xiao. Data curation, formal analysis, and investigation: Ang Li and Xiaolong Yang. Data curation, formal analysis, investigation, and funding acquisition: Hongze Chang. Project administration, supervision, and writing–review and editing: Feng Cai. Conceptualization, funding acquisition, project administration, supervision, and writing–review and editing: Xiaodong Liu.

## Funding

This work was supported by the Shanghai Natural Science Foundation (25ZR1401326), the Shanghai Healthcare System Key Discipline Construction Program (2024ZDXK0041), the Shanghai Rising‐Star Program (23YF1441400), and the Shanghai Yangpu District Science and Technology Commission and Health Commission Program (YPM202302).

## Conflicts of Interest

The authors declare no conflicts of interest.

## Supporting Information

Additional supporting information can be found online in the Supporting Information section.

## Supporting information


**Supporting Information** Supporting File 1: The complete search formula of all literature.

## Data Availability

The original data are available in both the article/supporting information, and the database‐related information is in the supporting information named “data,” further inquiries can be directed to the corresponding authors.
