# Supplementary material for: Research Hotspots and Emerging Trends in Osteoporosis Epigenetics
Source: Genet Res (Camb). 2026 May 20;2026:7389868. doi: 10.1155/genr/7389868 (PMC13189465; doi:10.1155/genr/7389868)
Supplement: Supplementary file 1 — Supporting Information Supporting File 1: The complete search formula of all literature. [file GENR-2026-7389868-s001.docx]

**Supplementary File 1:** The complete search formula of all literature.

TS=(Epigenomics OR Epigenomic OR Epigenetics OR Epigenetic OR “DNA Methylation” OR “DNA Methylations” OR “Methylation, DNA” OR “Methylations, DNA” OR “Histone Acetylation” OR “Histone Methylation” OR “Histone Modification” OR “Histone Ubiquitination” OR “Histone Ubiquitylation” OR “Histone Sumoylation” OR “Histone Sumoylations” OR “Histone Sumo Conjugation” OR “Histone Sumo-Conjugations” OR “Histone ADP-Ribosylation” OR “Histone Variants” OR “Histone H2a.F” OR “RNA Methylation” OR “RNA modification” OR “Chromatin Assembly” OR “Chromatin Assembly and Disassembly” OR “Chromatin Remodeling” OR Epitranscriptomic OR Epitranscriptomics OR “RNA, Untranslated” OR “Untranslated RNA” OR npcRNA OR “RNA, Nontranslated” OR “Nontranslated RNA” OR “RNA, Non-Peptide-Coding” OR “Non-Peptide-Coding RNA” OR “RNA, Non Peptide Coding” OR “RNA, Non-Protein-Coding” OR “Non-Protein-Coding RNA” OR “RNA, Non Protein Coding” OR “RNA, Noncoding” OR “Noncoding RNA” OR “RNA, Non-Coding” OR “Non-Coding RNA” OR “RNA, Non Coding” OR “RNA, Small Nuclear” OR snRNA OR “Small Nuclear RNA” OR “Low Molecular Weight Nuclear RNA” OR “Small Molecular Weight RNA” OR “Chromatin-Associated RNA” OR “Chromatin Associated RNA” OR “RNA, Chromatin-Associated” OR “RNA, Small Nucleolar” OR” Small Nucleolar RNA” OR snoRNA OR “telomerase RNA” OR TERC OR “TERC RNA, human” OR “telomerase RNA component, human” OR bTR OR “bovine telomerase RNA” OR “telomerase RNA component” OR “TERC RNA” OR “hTR RNA” OR MicroRNAs OR MicroRNA OR miRNAs OR miRNA OR “Primary MicroRNA” OR “MicroRNA, Primary” OR “Primary miRNA” OR “miRNA, Primary” OR pri-miRNA OR “RNA, Small Temporal” OR “Temporal RNA, Small” OR stRNA OR “Small Temporal RNA” OR pre-miRNA OR “RNA, Small Interfering” OR “Interfering RNA, Small” OR “Short Interfering RNA” OR “Interfering RNA, Short” OR “RNA, Short Interfering” OR “Small Interfering RNA” OR siRNA OR “Piwi-Interacting RNA” OR “Piwi Interacting RNA” OR “RNA, Piwi-Interacting” OR “RNA, Piwi Interacting” OR piRNA OR “Short Hairpin RNA” OR “Hairpin RNA, Short” OR “RNA, Short Hairpin” OR “Small Hairpin RNA” OR “Hairpin RNA, Small” OR “RNA, Small Hairpin” OR shRNA OR “Repeat-Associated siRNA” OR “Repeat Associated siRNA” OR “siRNA, Repeat-Associated” OR “siRNA, Repeat Associated” OR “Scan RNA” OR scnRNA OR “Small Scan RNA” OR “RNA, Small Scan” OR “Scan RNA, Small” OR “RNA, Scan” OR “Trans-Acting siRNA” OR “Trans Acting siRNA” OR tasiRNA OR “siRNA, Trans-Acting” OR “siRNA, Trans Acting” OR “RNA, Long Noncoding” OR “Noncoding RNA, Long” OR lncRNA OR “Long ncRNA” OR “ncRNA, Long” OR “RNA, Long Non-Translated” OR “Long Non-Translated RNA” OR “Non-Translated RNA, Long” OR “RNA, Long Non Translated” OR “Long Non-Coding RNA” OR “Long Non Coding RNA” OR “Non-Coding RNA, Long” OR “RNA, Long Non-Coding” OR “Long Non-Protein-Coding RNA” OR “Long Non Protein Coding RNA” OR “Non-Protein-Coding RNA, Long” OR “RNA, Long Non-Protein-Coding” OR “Long Noncoding RNA” OR “RNA, Long Untranslated” OR “Long Untranslated RNA” OR “Untranslated RNA, Long” OR “Long ncRNAs” OR “ncRNAs, Long” OR “Long Intergenic Non-Protein Coding RNA” OR “Long Intergenic Non Protein” OR “Coding RNA” OR LincRNA OR “LINC RNA” OR LincRNAs OR “RNA, Circular” OR circRNAs OR “Closed Circular RNA” OR “Circular RNA, Closed” OR “RNA, Closed Circular” OR “Circular RNA” OR “Circular RNAs” OR “RNAs, Circular” OR circRNA OR “Circular Intronic RNA” OR “Intronic RNA, Circular” OR “RNA, Circular Intronic” OR ciRNA) AND TS=(Osteoporoses OR “Osteoporosis, Age-Related” OR “Osteoporosis, Age Related” OR “Age-Related Osteoporosis” OR “Age-Related Osteoporoses” OR “Age Related Osteoporosis” OR “Osteoporoses, Age-Related” OR “Bone Loss, Age-Related” OR “Age-Related Bone Loss” OR “Age-Related Bone Losses” OR “Bone Loss, Age Related” OR “Bone Losses, Age-Related” OR “Osteoporosis, Senile” OR “Osteoporoses, Senile” OR “Senile Osteoporoses” OR “Senile Osteoporosis” OR “Osteoporosis, Involutional” OR “Osteoporosis, Post-Traumatic” OR “Osteoporosis, Post Traumatic” OR “Post-Traumatic Osteoporoses” OR “Post-Traumatic Osteoporosis” OR “Osteoporosis, Post-Menopausal” OR “Osteoporoses, Post-Menopausal” OR “Osteoporosis, Post Menopausal” OR “Post-Menopausal Osteoporoses” OR “Post-Menopausal Osteoporosis” OR “Postmenopausal Osteoporosis” OR “Osteoporoses, Postmenopausal” OR “Postmenopausal Osteoporoses” OR “Perimenopausal Bone Loss” OR “Bone Loss, Postmenopausal” OR “Bone Losses, Postmenopausal” OR “Postmenopausal Bone Losses” OR “Postmenopausal Bone Loss” OR “Bone Loss, Perimenopausal” OR “Bone Losses, Perimenopausal” OR “Perimenopausal Bone Losses”)
